# Medical Clowning: A Cost-Effective Way to Reduce Stress Among Children Undergoing Invasive Procedures

**DOI:** 10.7759/cureus.18886

**Published:** 2021-10-19

**Authors:** Tahleel Javed, Armughan S Khan, Nafees A Jarral, Zara Taqi, Maryum Raza, Zarmeen Shahid

**Affiliations:** 1 Medicine, Foundation University Medical College, Islamabad, PAK

**Keywords:** primary care medicine, complementary medicine, medicine, stress, medical clowning, anxiety

## Abstract

Background

Distraction techniques like medical clowning and the use of soap bubbles can aid in reducing children’s stress levels while undergoing invasive medical procedures. Such complementary therapies are not a common practice in Pakistan, and data exploring the potential benefits of complementary therapies are sparse. This study aimed to determine whether distractions like medical clowns and soap bubbles could reduce anxiety and pain perceived by children undergoing invasive medical procedures in a hospital in Pakistan.

Material and methods

We conducted a randomized controlled trial of 76 pediatric patients (aged six to 12 years) whose treatment required a peripheral intravenous (IV) catheter insertion at the pediatric ward of the Fauji Foundation Hospital in Rawalpindi, Pakistan, from March 2016 to June 2016. Peripheral IV catheter insertion was required for all patients as part of their treatments (no participants received IV catheter placement solely for this study). Our sample size was selected via random sampling, and we excluded patients whose parents or legal guardians did not consent for their inclusion. Study participants were randomly assigned to either a clown group (n=38) or a control group (n=38). The patients in the clown group underwent IV catheter placement while interacting with the medical student clown and soap bubbles in the presence of a parent. Patients in the control group underwent IV catheter placement with support provided only by the parent. We assessed the patient’s distress and anxiety before, during, and after the procedure. We used the Observation Scale of Behavioral Distress (OSBD), before and after the procedure with the short version of self-reported Spielberger’s State-Trait Anxiety Inventory-Children (STAI-C), the visual analog anxiety scale (VAS), and pain experienced with the Wong-Baker Faces pain scale (FPS) only after the procedure. Additionally, we collected demographic information. The hospital's ethical review committee approved our study design.

Results

Of the 76 study participants, 53.9% were male and 46.1% were female. Most patients lived in a rural setting (67%). Mean values of the FPS, OSBD, and STAI-C for the clown group (3.21, 6.23, and 8.52, respectively) were all lower than those for the control group (8.00, 18.02, and 15.29, respectively; p<.001); however, the difference was not statistically significant for children older than 10 years. After IV catheter placement, the mean VAS score for the clown group was also significantly lower than that for the control group (2.84 vs. 8.92, respectively; p<.001).

Conclusion

The use of distractions via medical clowns and soap bubbles was an effective nonpharmacological method of reducing anxiety and perceived pain in children undergoing invasive medical procedures. Therefore, proceduralists could use such techniques as powerful, noninvasive, and cost-effective complementary and alternative medicine tools in pediatric treatment settings in Pakistan. Further studies on the potential benefits of the aforementioned techniques are warranted.

## Introduction

Children generally have higher levels of fear and lower pain threshold levels than adults for injection and venipuncture [1-4], and more than 90% of children admitted to a hospital for care undergo a distressing procedure, such as injections and peripheral intravenous (IV) catheter placements [5]. The insertion of an IV catheter is one of the most common and painful invasive procedures in pediatric hospitals [6]. These experiences can lead to needle phobias and an aversion to seeking appropriate care at hospitals in the future [7,8]. Furthermore, although medical staff in pediatric departments spend a considerable amount of time desensitizing children toward such procedures to reduce psychologically induced changes like tachycardia, they often employ intrinsically stress-inducing methods such as restricting the child's physical movement [9].

Physicians and researchers have been interested in “medical clowning” and its therapeutic benefits in recent decades. A medical clown is a medical expert who is trained in the performing arts to help children cope with treatment and mitigate potential psychological or physical trauma through techniques such as humor, distraction, or misdirection. Hunter Doherty “Patch” Adams introduced medical clowning in 1971 at his Gesundheit! Institute in West Virginia. Through Adams’s advocacy and passion, medical clowns began operating across America in the late 1980s. Currently, various countries have embraced the idea of medical clowning and integrated the practice into their health care models [10].

However, given that it has never been researched or widely applied in this population, the potential benefits of humor in health care are somewhat alien in Pakistan. Therefore, the primary objective of this study was to determine the existence and magnitude of the effect of medical clowning (including the use of soap bubbles) on Pakistani children’s anxiety levels during the common invasive medical procedure of peripheral IV catheter placement. The secondary objective of this study was to determine if perceived pain levels in children undergoing the procedure were affected by the use of medical clowning.

## Materials and methods

We conducted a randomized controlled trial (RCT) of young patients (i.e., children aged six to 12 years) presenting to the pediatric ward of the Fauji Foundation Hospital (FFH) in Rawalpindi, Pakistan, from March 2016 to June 2016. Participants were eligible for inclusion if their treatment required insertion of a peripheral IV catheter (no participants received IV catheter placement for the sole purpose of this study). Participants, parents, and/or legal guardians who met the study criteria were included on a voluntary basis after providing written informed consent to participate in the study. While informing potential participants on the procedure, we highlighted that clowning techniques would be used. The study excluded patients, parents, or legal guardians of patients who did not consent to their inclusion or expressed any feelings of unease regarding clowns (including coulrophobia/fear of clowns). The hospital's ethical review committee approved our study design.

We used a sample size calculator to determine an appropriately sized population for an adequate RCT [11] at a 95% confidence level (n=76). Once we enrolled 76 patients based on the availability and inclusion criteria, participants were randomly and evenly distributed between a test group (medical clowning, n=38) and a control group (no medical clowning, n=38). The study intervention was limited to the procedure room of the pediatric ward to avoid any potential impact the presence of a clown may have on nonparticipating patients. In the clown group, the patient underwent IV catheter placement while interacting with the medical student clown and soap bubbles in the presence of a parent. The medical clowns put on their costumes in an allocated corner of the procedure room, which was covered by a curtain. After the intervention, the medical clowns were instructed to change out of their costumes before leaving the procedure room. Patients in the control group underwent IV catheter placement with support provided by the parent, with no medical clown present.

We assessed the patient’s distress and anxiety before, during, and after the procedure. We used the Observation Scale of Behavioral Distress (OSBD) [12], before and after the procedure with the short version of self-reported Spielberger’s State-Trait Anxiety Inventory-Children (STAI-C) [13], the visual analog anxiety scale (VAS) [14], and pain experienced with the Wong-Baker Faces Pain Scale (FPS) after the procedure [15,16]. Additionally, we collected gender and sociodemographic information for each participant. We used IBM SPSS Statistics for Windows, version 21.0 (IBM Corp., Armonk, NY) for statistical analysis of the data.

## Results

**Figure 1:**
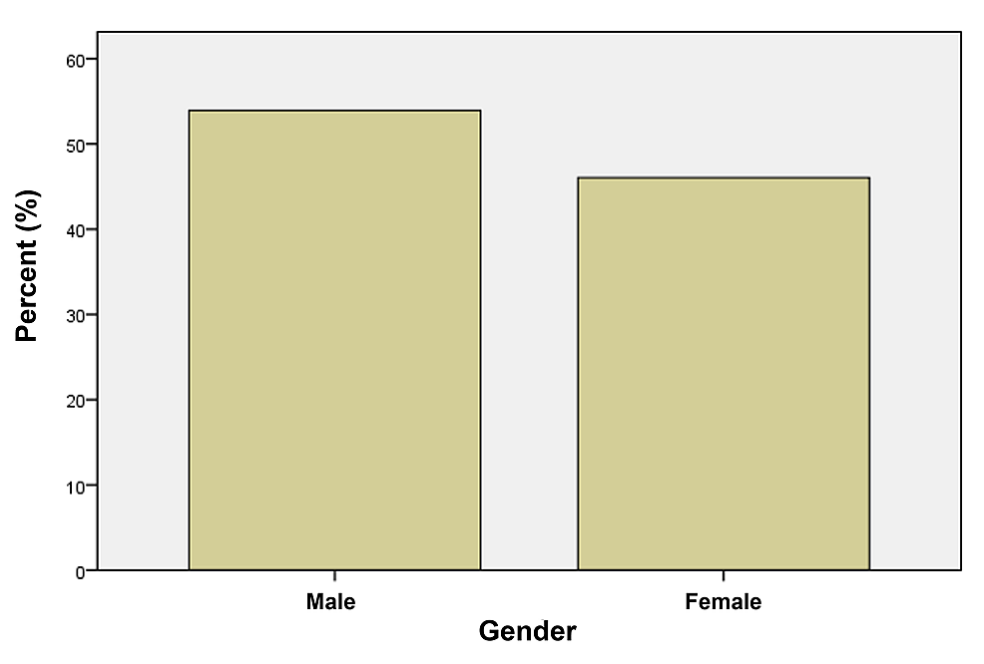
Gender distribution of the study population

**Figure 2:**
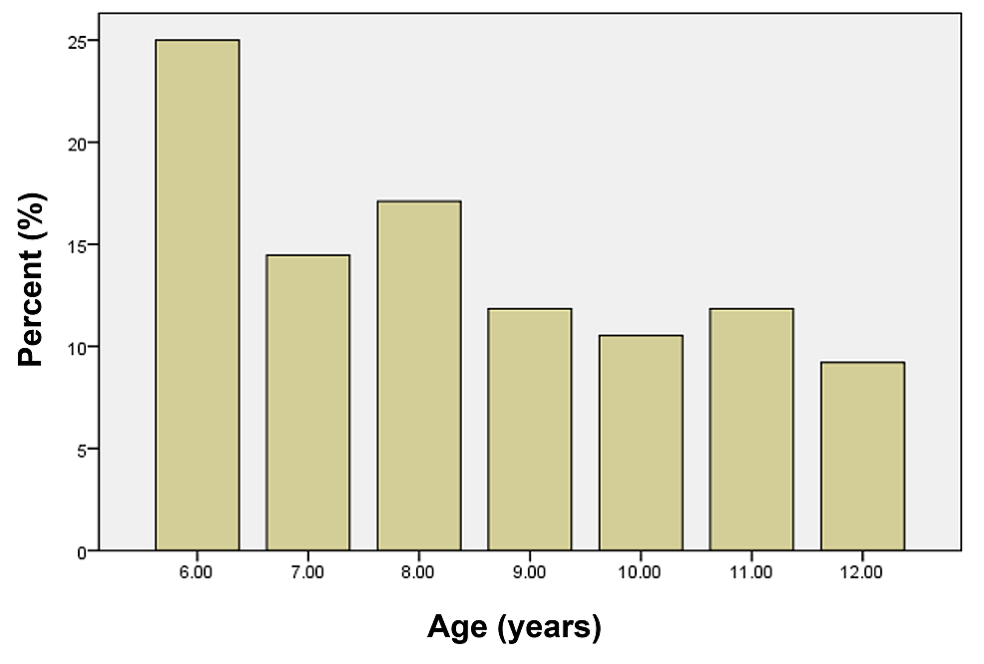
Age distribution of the study population

Among the 76 study participants, 53.9% were male, and 46.1% were female patients (Figure 1). Six-year-old patients comprised the largest age group in the study (Figure 2), and the majority of patients lived in a rural setting (67%), with 23% living in urban and 10% in suburban settings of the Punjab province of Pakistan.

**Figure 3:**
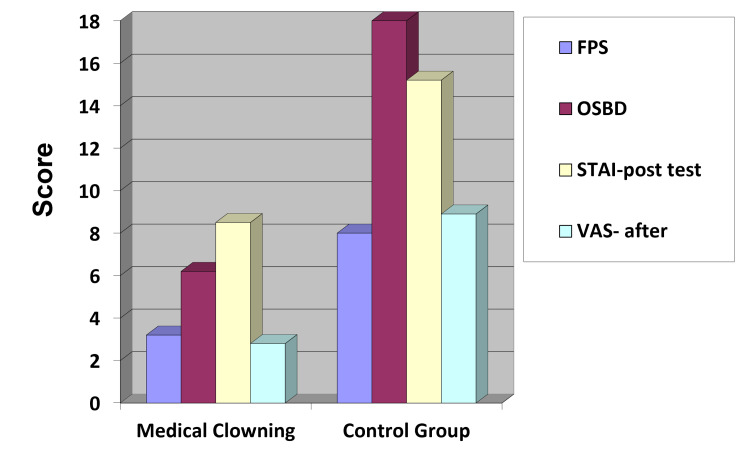
Mean FPS, OSBD, STAI, and VAS of test and control group FPS: Wong-Baker Faces Pain Scale, OSBD: Observation Scale of Behavioral Distress, STAI: Spielberger's State-Trait Anxiety Inventory, VAS: Visual Analog Anxiety Scale.

Mean values of the FPS, OSBD, and STAI-C for the clown group (3.21, 6.23, and 8.52, respectively) were all lower than those for the control group (8.00, 18.02, and 15.29, respectively; p<.001; Figure 3; Tables 1, 2); however, the difference was not statistically significant for children older than age 10. Following the procedure, the mean value of the VAS for the clown group was also significantly lower than the control group’s mean value (2.84 vs. 8.92, respectively; p<.001). Patient gender and socioeconomic status had no impact on the study results or the nature of the procedure. 

 

**Table 1:** Comparison of pain and anxiety scores in children undergoing invasive medical procedure with medical clowning and without medical clowning FPS: Wong-Baker Faces Pain Scale, OSBD: Observation Scale of Behavioral Distress, SE: standard error, SD: standard deviation, STAI: Spielberger’s State-Trait Anxiety Inventory, VAS: Visual Analog Anxiety Scale.

Evaluation	Clowning	N	Mean	SD	SE Mean
FPS	Present	38	3.21	2.16	.35
	Absent	38	8.00	2.03	.33
OSBD	Present	38	6.24	4.73	.77
	Absent	38	18.03	4.67	.76
STAI (post)	Present	38	8.53	2.15	.35
	Absent	38	15.29	2.54	.41
VAS (before)	Present	38	7.26	1.55	.25
	Absent	38	5.97	1.72	.28
VAS (after)	Present	38	2.84	1.79	.29
	Absent	38	8.92	1.44	.23

**Table 2:** Independent samples t-test F: test statistic of Levene's test, SE: standard error, Sig.: significance, t: computed test statistic, df: degrees of freedom, FPS: Wong-Baker Faces Pain Scale, OSBD: Observation Scale of Behavioral Distress, STAI: Spielberger’s State-Trait Anxiety Inventory, VAS: Visual Analog Anxiety Scale.

		Levene's Test for Equality of Variances		t-test for Equality of Means							
									95% Confidence Interval of the Difference		
		F	Sig.	t	df	Sig. (two-tailed)	Mean Difference	SE Difference	Lower	Upper	
FPS	Equal variances assumed	.540	.465	-9.972	74	.000	-4.78947	.48028	-5.74645	-3.83250	
	Equal variances not assumed			-9.972	73.711	.000	-4.78947	.48028	-5.74651	-3.83244	
OSBD	Equal variances assumed	.452	.503	-10.937	74	.000	-11.78947	1.07798	-13.93740	-9.64154	
	Equal variances not assumed			-10.937	73.989	.000	-11.78947	1.07798	-13.93741	-9.64154	
STAI (post)	Equal variances assumed	1.115	.294	-12.535	74	.000	-6.76316	.53954	-7.83821	-5.68810	
	Equal variances not assumed			-12.535	72.106	.000	-6.76316	.53954	-7.83868	-5.68763	
VAS (before)	Equal variances assumed	.212	.646	3.433	74	.001	1.28947	.37559	.54109	2.03785	
	Equal variances not assumed			3.433	73.284	.001	1.28947	.37559	.54097	2.03798	
VAS (after)	Equal variances assumed	2.849	.096	-16.289	74	.000	-6.07895	.37319	-6.82255	-5.33535	
	Equal variances not assumed			-16.289	70.702	.000	-6.07895	.37319	-6.82312	-5.33477	

## Discussion

This study is the first of its kind to be conducted in Pakistan to determine whether the presence of distractions like medical clowns and soap bubbles can reduce anxiety and perceived pain in children undergoing invasive medical procedures. In general, medical care in Pakistan has not formally incorporated medical clowning as a therapeutic supplement, and this study highlights the potential benefit of incorporating such approaches.

Pain and anxiety associated with health care can have long-term adverse effects on patient's well-being and care-seeking behavior. Painful memories from unsuccessful pain relief efforts in children cause immense voluntary reactions (e.g., crying and avoiding injections) and psychological responses (e.g., tachycardia and vascular spasm) in subsequent experiences [17]. Hindering physical movement is a common practice by nurses to administer an injection, further aggravating children’s fear and retaliation response. Such methods can amplify distress associated with hospitals and cause communication and trust issues for the young patient (e.g., “needle phobia”) [17]. This can traumatize the child and increase the difficulty for the health care provider in treating the patient. Over time, these adverse experiences can lead to a patient preferring to hide an illness and suffer without treatment rather than going to the hospital [8].

Distraction therapy

Distraction has been studied as a possible approach to help alleviate pain and anxiety in patients. Caprilli et al. conducted a prospective study on the effects of interactive music as supplemental therapy for pain and stress in 108 children (age range: four to 13 years) [18]. The authors reported reduced anxiety in the test group compared to the control, which aligned with our results.

Another Caprilli et al. distraction study evaluated soap bubbles as a means of patient distraction and their effect on pain and distress in children undergoing blood sampling [19]. They measured distress via OSBD and perceived pain using FPS. The correlation between distress during blood sampling and children’s age (r=-.571; p=.001) and the correlation between age and pain (r=-.577; p=.001) were significant. Our study found significant reductions in measured distress and perceived pain for children younger than age 10 using similar scales (p<.001 using an independent sample t-test).

Medical clowning as a distraction

Distraction via medical clowning can mitigate fears and anxiety in children. A study in France on medical clown use reported that despite the various obstacles faced to the practice, incorporating medical clowning was a worthwhile exercise [20]. A series of three cases in Israel assessed the use of medical clowning during the examination of sexually abused children. Medical clowning and the application of humor had significant anxiety-lowering effects on the traumatized children and helped the child overcome bad memories of the abuse during the forensic examination [21]. A 2016 study compared the effect of local anesthetic cream use during blood sampling in children with clowning on anxiety and pain levels. The authors found that the application of anesthetic cream reduced pain but that medical clowning resulted in even lower levels of anxiety and crying in children [22]. Another RCT evaluated medical clowning effect on anxiety and pain perceived by children undergoing the allergy skin prick test [23]. The authors found a significant reduction in anxiety using the STAI-C, supporting our findings. Another randomized prospective pilot study on the effect of a medical clown on pain and anxiety during IV access in the pediatric emergency department [24] using the FPS and VAS found a significant reduction in pain and anxiety, results similar to our findings.

Humor and medical treatment

Used as an adjunctive intervention in conventional medical practices, humor relieved fear and anxiety surrounding painful, invasive procedures in pediatric patients [20,25]. One successful large-scale implementation of this approach is found in Red Noses International, an institution of highly skilled physicians who provide a sense of reassurance and psychological support to their patients [26]. In France and most of Eastern Europe, medical clowning has been integrated as standard practice in pediatric health care. Medical clowning is not a means to avoid treatment; instead, it allows children a sense of harmony in the hospital environment [27]. Humor suppresses patient and parent anxiety and creates a lasting beneficial effect for hospital staff. Humor cultivates teamwork, improves morale and motivation, increases productivity, relaxes people, enhances problem-solving abilities, and creates a positive work culture with greater job satisfaction [28].

Similar studies should be conducted on a larger Pakistani population, given our results and the potential benefits of medical clowning for pediatric patients. Future studies should explore the efficacy of medical clowning techniques and their effects on children from various cultural backgrounds and environments within Pakistan, where medically proven complementary therapies are novel.

Limitations

Our relatively small population limited this study due to the availability and eligibility of the participants. Secondly, the study was conducted in the procedure room of only one tertiary care hospital.

## Conclusions

Medical clowning and soap bubble distractions were effective nonpharmacological methods of reducing anxiety and perceived pain in children undergoing cannulation. Policymakers and medical leadership in Pakistan should consider the potential benefits to pediatric patients that arise from medical clowning when treating children. Further studies are warranted to explore adopting this potentially potent, noninvasive, and cost-effective tool as a complementary therapy in children receiving medical treatment.
